# Inhibition of Myocardial Remodeling and Heart Failure by Traditional Herbal Medications: Evidence from Ginseng and *ginkgo biloba*

**DOI:** 10.31083/j.rcm2407212

**Published:** 2023-07-21

**Authors:** Morris Karmazyn, Xiaohong Tracey Gan

**Affiliations:** ^1^Department of Physiology and Pharmacology, University of Western Ontario, London, ON N6A 5C1, Canada

**Keywords:** ginseng/ginsenosides, ginkgo/ginkgolides, myocardial hypertrophy, myocardial remodeling, heart failure

## Abstract

Herbal-based medications have been used as therapeutic agents for thousands of years, particularly in Asian cultures. It is now well established that these herbal medications contain potent bioactive phytochemicals which exert a plethora of beneficial effects such as those seen on the cardiovascular system. Among the most widely studied of these herbal agents is ginseng, a member of the genus *Panax*, which has been shown to produce beneficial effects in terms of reducing cardiac pathology, at least in experimental studies. The beneficial effects of ginseng observed in such studies are likely attributable to their constituent ginsenosides, which are steroid-like saponins of which there are at least 100 and which vary according to ginseng species. Many ginseng species such as *Panax ginseng* (also known as Asian ginseng) and *P quinquefolius* (North American ginseng) as well as specific ginsenosides have been shown to attenuate hypertrophy as well as other indices of myocardial remodeling in a wide variety of experimental models. *Ginkgo biloba* on the other hand has been much less studied although the leaf extract of the ancient ginkgo tree has similarly consistently been shown to produce anti-remodeling effects. Ginkgo’s primary bioactive constituents are thought to be terpene trilactones called ginkgolides, of which there are currently seven known types. Ginkgo and ginkgolides have also been shown to produce anti-remodeling effects as have been shown for ginseng in a variety of experimental models, in some cases via similar mechanisms. Although a common single mechanism for the salutary effects of these compounds is unlikely, there are a number of examples of shared effects including antioxidant and antiapoptotic effects as well as inhibition of pro-hypertrophic intracellular signaling such as that involving the calcineurin pathway which results in the upregulation of pro-hypertrophic genes. Robust clinical evidence represented by large scale phase 3 trials is lacking although there is limited supporting evidence from small trials at least with respect to ginseng. Taken together, both ginseng and ginkgo as well as their bioactive components offer potential as adjuvant therapy for the treatment of myocardial remodeling and heart failure.

## 1. Introduction

**Fig. 1.**
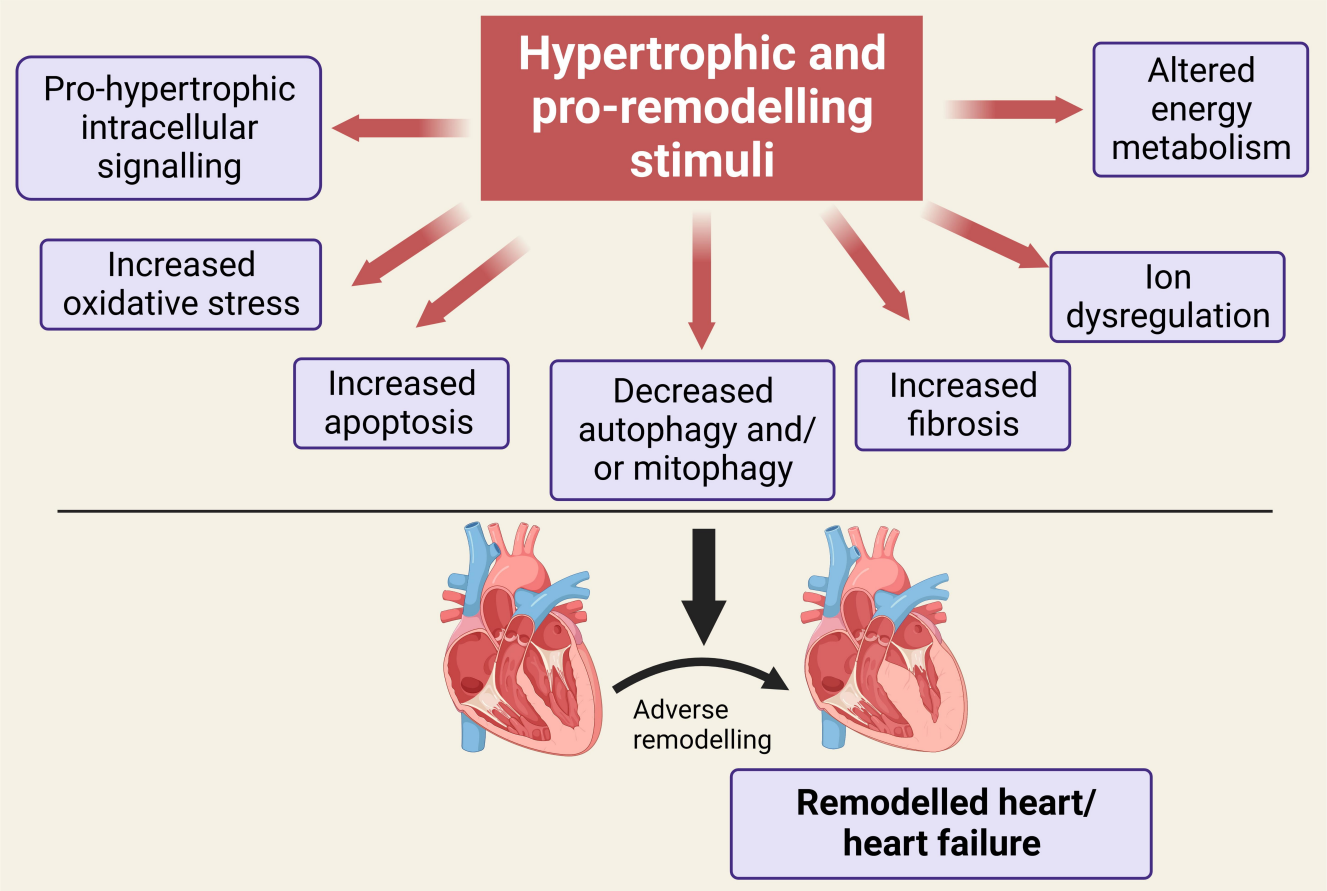
**Illustration showing a number of the key mediators of the remodeling process leading to heart failure following initial stimulation and which have been shown to be targeted by ginseng and ginkgo or their bioactive constituent components**. See text for details. Created with BioRender.com.

Heart failure represents a major medical challenge of the twenty first century. It is estimated that more than 64 million individuals are currently living with heart failure in the world today with numbers expected to rise substantially in the coming years. There has been substantial progress in the development of pharmacological agents and other approaches aimed at slowing heart failure progression including angiotensin-converting enzyme inhibitors or angiotensin receptor blockers, beta-blockers, diuretics and others [1]. Non-traditional pharmacological interventions have also been recently proposed. Among the most promising are the sodium-glucose co-transporter 2 inhibitors (“gliflozins”) initially introduced for the management of type 2 diabetes but which appear to have salutary effects for the treatment of heart failure particularly with early administration, through mechanisms unrelated to their blood glucose lowering effects [2]. These drugs have also been shown to offer benefit for the treatment of heart failure with preserved ejection fraction [3]. Nonetheless, mortality rates for heart failure remain high with 50% of patients dying within five years after the first diagnosis [4]. These high mortality rates likely reflect the complexity of the heart failure process following initial insult or injury to the myocardium which is followed by myocardial remodeling involving hypertrophy and myocardial fibrosis. Some of these main contributors to myocardial remodeling and heart failure are summarized in Fig. 1, which also represents potential sites of action of ginseng and ginkgo as well as their active constituents. One of the principal factors underlying the increased incidence of heart failure reflects the relative success in reducing deaths from myocardial infarction as many of these surviving patients eventually progress to heart failure initiated by the initial infarct. As most patients are diagnosed with heart failure after the remodeling process has already progressed, treatment is challenging as the most effective approach would be to reverse the myocardial remodeling process [5, 6].

The challenge to effectively treat heart failure has led to the identification of natural products as potential therapies for heart failure particularly as adjunctive treatments in concert with standard heart failure medications. Indeed, as will be discussed below, many of these natural compounds, such as ginseng, have a long history of use in Asian societies for thousands of years. The goal of this review is to discuss the potential efficacy of two natural approaches based on ancient Chinese medicines, namely ginseng and the less studied *ginkgo biloba* for treating heart failure and the underlying mechanisms for their effects.

## 2. Antihypertrophic Effects of Ginseng and Ginsenosides 

Among the most widely studied natural products with potential application for treating heart failure is ginseng, an ancient perennial herb belonging to the family *Araliaceae* and genus *Panax* which has an extensive history as a therapeutic agent particularly in Asian cultures. It is believed that the first use of ginseng as a medicinal product occurred in China during the Han Dynasty, between 206 BC and 220 AD [7]. It is important to note that ginseng is not a single entity but instead consists of hundreds of bioactive ingredients contributing to its therapeutic properties. Indeed, ginseng can contain up to 200 active ingredients depending on the ginseng type with the major active components being the saponin ginsenosides of which there are more than 50 found in virtually all components of the ginseng plant. Based on their structures, these ginsenosides are classified into three groups: the panaxatriol ginsenosides (Re, Rf, Rg1, Rg2, and Rh1), the panaxadiol ginsenosides (Rb1, Rb2, Rb3, Rc, Rd, Rg3, and Rh2), components of the dammarane type ginsenosides as well as oleanolic acid [8, 9]. As discussed in section 2.1, many of these ginsenosides have been found to exert antihypertrophic and anti-remodeling effects rendering them potential candidates for the treatment of heart failure. Adding to the complexity of ginseng is the fact that there are a variety of ginseng species which originate from different international regions with each possessing distinct chemical profiles such as North American (*Panax quinquefolius*) and Asian ginseng (*P ginseng*, *P notoginseng*). These are generally referred to as white ginseng although subjecting these white ginseng varieties to heating protocols converts them to red ginseng (also referred to as Korean ginseng) which alters their chemical profiles in terms of both ginsenoside and non-ginsenoside components thus potentially enhancing their efficacy [7, 10].

There are substantial data from animal research demonstrating that ginseng-related products as well as its constituent ginsenosides exert potent antihypertrophic effects based on studies using both *in vitro* and *in vivo* experimental approaches. With respect to the former, it has been shown that trilinolein, an extract of *P notoginseng*, a widely used Chinese medication, effectively prevented the hypertrophic effect produced by angiotensin II [11], norepinephrine [12] and endothelin-1 [13, 14] when administered to cultured neonatal rat ventricular myocytes. In all studies using trilinolein as the antihypertrophic agent, the salutary effects were attributed to an antioxidant property [11, 12, 13, 14]. A *P notoginseng* extract was further shown to reduce heart failure in rats subjected to myocardial infarction through a mechanism involving the activation of the transcriptional factor peroxisome proliferator-activated receptor α (PPARα) which is intimately involved in the regulation of energy metabolism [15]. Moreover, a total saponin extract of *P notoginseng* effectively reduced heart failure in mice subjected to chronic coronary artery ligation through a mechanism involving enhanced autophagy in the hearts of these animals [16]. This enhanced autophagy may be closely related to the generation of endoplasmic reticulum [ER] stress. In this regard, it was recently shown that *P notoginseng* prevents ER stress produced by administration of thapsigargin in neonatal rat ventricular myocytes [17]. Moreover, prevention of autophagy in H9c2 myoblasts abolishes Panax Notoginseng Saponins (PNS) protection against thapsigargin-induced ER stress response as well as the accompanying apoptosis [17].

While most studies used root extracts from ginseng species, floral extracts of *P notoginseng* have also been shown to exert antihypertrophic effects. Thus, a *P notoginseng* flower extract administered to chymase-overexpressing transgenic mice effectively reduced the resultant hypertrophic and remodeling effects through a mechanism associated with reduced expression of chymase, transforming growth factor beta (TGF-β1), Smad2 and Smad3 while upregulating expression levels of Smad7 [18]. *P notoginseng*, therefore, appears to be an effective antihypertrophic agent acting primarily through its constituent notoginsenosides. The effects of individual ginsenosides are discussed in chapter 2.3, however it is relevant to note at this point that notoginsenoside R1, a unique constituent of *P notoginseng*, has been shown to reduce hypertrophic responses in various experimental models such as isoproterenol-treated atherosclerosis-prone mice, through a mechanism potentially involving a reduction in the inflammatory response [19]. Moreover, this ginsenoside was further shown to reduce diabetic cardiomyopathy in *db/db* diabetic mice potentially by inhibiting oxidative stress and apoptosis in the hearts of these animals via the upregulation of cardiac estrogen α receptor expression which in turn led to the activation of Akt-Nrf2 (Ak strain transforming (protein kinase B)-nuclear factor erythroid 2-related factor 2) signaling as well as inhibition of the TGFβ pathway [20]. The beneficial effects of notoginsenoside R1 were also evident in H9c2 myoblasts treated with advanced glycation end products, or advanced glycation end products (AGEs), which produce various pathologies associated with diabetes mellitus [20].

Recent evidence further suggests that *P notoginseng*-containing medications with a history of use in China can reduce heart failure in a variety of experimental models. Among these include DanQi Pill (DQP) derived from both Salvia miltiorrhiza, a popular medicinal herb in Asian countries as well as *P notoginseng*, a medicinal which has been and continues to be used in China particularly for the treatment of myocardial infarction and heart failure. Experimental studies show that DQP can improve cardiac function and reduce hypertrophy in a rat heart failure model secondary to coronary artery ligation although the precise mechanism underlying these effects is not precisely known. One study implicated a reduction in pro-inflammatory eicosanoids including leukotrienes as the primary mechanism [21]. Using a similar model it has recently been proposed that the protective effect of DQP against heart failure post infarction was mediated by increased mitophagy [22], a process which involves selective autophagic removal of dysfunctional mitochondria which functions as a cardiac self-protective mechanism [23].

### 2.1 P ginseng

*P ginseng* or Asian ginseng has also been shown to reduce heart failure such as that produced by administering the anticancer cardiotoxic drug Adriamycin possibly via an antioxidant influence [24].

### 2.2 P quinquefolius

*P quinquefolius*, or North American ginseng (NAG) grown in various regions of Canada and the United States, has received substantial attention as a potential treatment for reducing hypertrophy, myocardial remodeling and heart failure in a number of experimental models. Numerous ginsenosides have been identified in NAG with ginsenoside Rb1 appearing to be most abundant with very low levels of Rb3 [25]. For more extensive discussion of NAG, a detailed review of the bioactive constituents of NAG and their general extensive pharmacological profiles has recently been published [26]. Our laboratory has carried out extensive studies to determine whether NAG exerts beneficial effects in a variety of experimental models of hypertrophy and heart failure. Among our findings was the observation that NAG reduced the hypertrophic response in cultured neonatal rat ventricular myocytes treated for 24 hours with the $\alpha_{1}$ adrenoceptor agonist phenylephrine [27]. Moreover, NAG suppressed both the hypertrophy as well as left ventricular dysfunction in rats subjected to 4 weeks of sustained coronary artery ligation (CAL) [27]. The beneficial effects of NAG in both the cultured myocytes as well as in animals subjected to CAL were associated with an inhibition in the upregulation of ${\mathrm{Na}}^{+}$-$H^{+}$ exchanger 1 (NHE-1) expression and activity concomitant with suppressed calcineurin activity as well as the transcriptional factors nuclear factor of activated T cells (NFAT) 3 and GATA-4 (GATA-binding protein 4) [27]. Moreover, administering NAG after 4 weeks of sustained coronary artery ligation when heart failure was already established provided substantial reduction in heart failure and myocardial remodeling when administered for a further 4-week period thus implicating an ability of NAG to reverse the remodeling process [28]. As noted above, reversal of myocardial remodeling and the resultant heart failure represent highly desirable, albeit elusive goals in heart failure therapeutics [5, 6]. At present reverse remodeling can be achieved primarily by mechanical unloading of the heart with the use of left ventricular assist devices [29]. The results with NAG suggest therefore that this ginseng species could be a very useful adjunctive therapy for treating heart failure when combined with standard heart failure medications.

We and others have also reported that NAG suppresses the hypertrophic responses and improves left ventricular function in other experimental models not related to coronary artery ligation. For example, NAG administration reduced hypertrophy and improved cardiac function in rats treated for two weeks with isoproterenol [30]. The beneficial effects of NAG were associated with inhibition of both protein kinase A and cAMP response element-binding protein phosphorylation [30]. The beneficial effects of NAG against isoproterenol induced hypertrophy and heart failure were similar to those seen with Ginsenoside Re, a primary ginsenoside component of *P ginseng *which has also been identified in NAG [31]. NAG exerted similar beneficial effects as that seen against isoproterenol-induced cardiac effects by inhibiting both hypertrophy and left ventricular dysfunction in rats treated for either 2 or 4 weeks with angiotensin II, effects associated with normalization in fatty acid and glucose oxidation [32]. Lastly, NAG was also found to effectively prevent the hypertrophic effects of the pro-satiety adipokine leptin in cultured neonatal rat ventricular myocytes through a mechanism involving inhibition of the p115Rho guanine nucleotide exchange factor-RhoA/Rho-associated, coiled-coil containing protein kinase-dependent mitogen-activated protein kinase (RhoA/ROCK) pathway [33].

### 2.3 Individual Ginsenosides as Antihypertrophic Agents

As is the case for all ginseng species, identifying the primary components underlying their beneficial effects represents a major challenge particularly because of the presence of numerous bioactive components in ginseng. In discussing the antihypertrophic effect of *P notoginseng* (section 2.1) it was noted that notoginsenoside R1 may account for the beneficial effect of *P notoginseng*. However, *P notoginseng* also contains a number of other ginsenosides in addition to notoginsenoside R1 including ginsenoside Rb1, ginsenoside Rd, ginsenoside Rg1 and ginsenoside Re [34]. Recently, using a mouse heart failure model and hypoxic-reoxygenated H9c2 cells, ginsenoside Rb3 was identified as potentially having the best pharmacodynamic profile in terms of exerting cardiac protection against indices of heart failure, exerting the salutary effects primarily via PPARα activation [35]. However, few studies have reported beneficial effects of ginsenoside Rb3 in terms of reducing heart failure, possibly due to its poor bioavailability following oral ingestion. Interestingly, conjugation of ginsenoside Rb3 to a nano carrier improved its bioavailability while also improving left ventricular function with reduced fibrosis when administered to rats subjected to 28 days of thoracic aortic banding [36]. These effects appeared to be related to PPARα as the conjugate prevented downregulation of PPARα expression in hearts of aortic banded rats [36]. Indeed, the overall benefit of the ginsenoside Rb3 conjugate was generally superior to the effects seen with the angiotensin AT1 receptor antagonist valsartan [36].

Other ginsenosides have similarly shown robust antihypertrophic properties. A total ginsenoside extract from *P ginseng* was shown to suppress right ventricular hypertrophy (RVH) in rats treated with monocrotaline, a pyrrolizidine alkaloid which produces RVH secondary to pulmonary hypertension [37]. The protection by the ginsenoside extract preparation was associated with an inhibition of the increased right ventricular myocardial expression of calcineurin as well as mitogen activated protein kinase in monocrotaline treated animals [37]. Similar results were seen with individual ginsenoside Rb1 which inhibited right ventricular hypertrophy in monocrotaline-treated rats [38]. These effects were associated with suppressed cardiac expression of calcineurin and the transcriptional factors NFAT3 and GATA-4 as described above for *P quinquefolius * [27]. Ginsenoside Rb1 also inhibited left ventricular dysfunction in a transgenic mouse model of dilated cardiomyopathy [39]. This ginsenoside also reduced hypertrophy and improved cardiac function in *db/db* diabetic mice treated for 12 weeks with Rb1, an effect attributed to inhibition in pro-inflammatory adipokines [40]. An anti-inflammatory effect was also proposed as the primary mechanism underlying the antihypertrophic effect of Rb1 in mice subjected to 14 days of angiotensin II infusion [41]. Further benefit of Rb1 was shown when administered to aged mice in which this ginsenoside was shown to reduce markers of inflammation and cardiac fibrosis, representing an important component of the myocardial remodeling process [42]. Inhibition of calcineurin and NFAT3/GATA-4 activation appears also to represent the primary mechanism underlying the antihypertrophic effects of Rb1 as shown in studies using cultured neonatal rat ventricular cardiomyocytes subjected to the pro-hypertrophic effect prostaglandin F2α [43].

Ginsenoside Rg1 has similarly been shown to produce an antihypertrophic effect when administered to rats subjected to 21 days of pressure overload using an aortic banding model. In that report, Rg1 significantly reduced left ventricular hypertrophy (LVH) through a mechanism proposed to be mediated by both the inhibition of the calcineurin/NFAT3/GATA-4 as well as mitogen activated protein kinase (MAPK) pathways [44] whereas a subsequent study by these authors using an identical experimental model proposed increased endogenous nitric oxide generation as the underlying mechanism [45]. Using a similar aortic banding model of LVH, Rg1 has also been reported to reduce hypertrophy through a mechanism involving reduced fibrosis and enhanced angiogenesis through increased expression of hypoxia-inducible factor-1 (HIF-1) and vascular endothelial growth factor (VEGF) [46]. The salutary effect of Rg1 was also associated with activation of phospho-Akt and inhibition of p38 MAPK [46]. Rg1 has also been shown to improve cardiac function in a streptozotocin rat diabetes model via a reduction in oxidative stress in these animals [47]. Recently, Rg1 was shown to reduce myocardial damage and improve cardiac function in mice subjected to 28 days coronary artery ligation via a mechanism involving increased mitophagy thus enhancing degradation of damaged mitochondria as well as reduced mitochondrial injury [48]. Similar protection of Rg1 was demonstrated in H9c2 cells treated with hydrogen peroxide which was associated with decreased apoptosis in these cells [48].

The major ginsenoside Rg2 was shown to reduce fibrosis and improve cardiac function when administered to mice subjected to two weeks of sustained coronary artery ligation, effects attributed to increased Akt phosphorylation [49]. Another ginsenoside shown to exert beneficial effects on myocardial remodeling and heart failure is ginsenoside Rg3, a major component found in various ginseng species. In this regard Rg3 reduced hypertrophy both in rats subjected to aortic banding as well as in a cardiac cell line treated with angiotensin II [50]. The underlying mechanisms were proposed to involve inhibition of NF-κB (nuclear factor kappa-light-chain-enhancer of activated B cells) activation and increased expression of sirtuin 1 thus reducing oxidative stress [50]. It has also been proposed that Rg3 reduces hypertrophy and improves left ventricular function in aortic banded mice by improving intracellular ${\mathrm{Ca}}^{2+}$ homeostasis through a mechanism involving enhanced SUMOylation of sarcoplasmic/endoplasmic reticulum ${\mathrm{Ca}}^{2+}$ ATPase 2a (SERCA2a) [51]. Ginsenoside Rd, found in NAG and to a lesser extent in *P ginseng*, has also been shown to exert antihypertrophic effects both *in vivo* as well as in isolated myocytes. Thus, this ginsenoside reduced hypertrophy in mice subjected to 2 weeks of aortic banding as well as in cultured cardiomyocytes treated with phenylephrine through mechanisms involving attenuation of oxidative stress, MAPK signaling pathway as well as the inflammatory response [52].

A summary of the effects of ginseng and ginsenosides and their proposed underlying mechanisms is presented in Table 1 (Ref. [11, 12, 13, 14, 15, 16, 18, 19, 20, 24, 27, 28, 30, 31, 32, 33, 35, 36, 37, 38, 39, 40, 41, 42, 43, 44, 45, 46, 47, 48, 49, 50, 51, 52]).

**Table 1.** **Studies demonstrating antiremodeling effects and reduction in heart failure by total ginseng extracts and individual ginsenosides in various experimental models and their proposed mechanisms of action**.

Ginseng or ginsenoside	Experimental model	Proposed mechanism(s)	Ref
*P notoginseng*	Ang II induced NRVM hypertrophy	antioxidant	[11]
	NE induced NRVM hypertrophy	antioxidant	[12]
	ET-1 induced NRVM hypertrophy	antioxidant	[13, 14]
	Rat CAL	PPARα activation	[15]
	Rat CAL	enhanced autophagy	[16]
	Chymase overexpressing mice	modulating the TGF-β/Smad pathway	[18]
*P ginseng*	Adriamycin treated rats	antioxidant	[24]
	Monocrotaline induced RVH in rats	calcineurin/MAPK inhibition	[37]
*P quinquefolius*	PE induced NRVM hypertrophy	NHE1 downregulation	[27]
	Rat CAL	NHE1 downregulation	[27]
	Rat CAL	calcineurin/NFAT3 inhibition	[28]
	Ang II induced NRVM hypertrophy	calcineurin/NFAT3 inhibition	[28]
	ET-1 induced NRVM hypertrophy	calcineurin/NFAT3 inhibition	[28]
	PE induced NRVM hypertrophy	calcineurin/NFAT3 inhibition	[28]
	Isoproterenol infusion in rats	decreased PKA and CREB phosphorylation	[30]
	ISO induced NRVM hypertrophy	decreased PKA and CREB phosphorylation	[30]
	Ang II infusion in rats	improved FA and glucose oxidation	[32]
	Leptin induced NRVM hypertrophy	RhoA/ROCK inhibition	[33]
Notoginsenoside R1	Isoproterenol infusion in mice	reduced inflammatory response	[19]
	*db/db* diabetic mice/AGE-treated H9c2 cells	activation of estrogen α receptor	[20]
Ginsenoside Re	Isoproterenol infusion in rats	decreased TGF-β1/Smad3	[31]
Ginsenoside Rb3	Mouse CAL	PPARα activation	[35]
	Rat TAB	PPARα activation	[36]
Ginsenoside Rb1	Monocrotaline induced RVH in rats	calcineurin/NFAT3/GATA-4 inhibition	[38]
	DCM in TG mice	STAT3 inhibition	[39]
	*db/db* diabetic mice	adipokine inhibition	[40]
	Ang II infusion in mice	decreased inflammatory response	[41]
	Aged mice	NF-κB modulation	[42]
	PGF2α induced NRVM hypertrophy	calcineurin/NFAT3/GATA-4 inhibition	[43]
Ginsenoside Rg1	Rat TAB	calcineurin/NFAT3/GATA-4 and MAPK inhibition	[44]
	Rat TAB	increased NO generation	[45]
	Rat TAB	increased HIF-1/VEGF expression, p-Akt activation and p38 MAPK inhibition	[46]
	STZ diabetic rat	reduced oxidative stress	[47]
	Mouse CAL	increased mitophagy	[48]
	Hydrogen peroxide treated H9c2 cells	reduced apoptosis	[48]
Ginsenoside Rg2	Mouse CAL	increased Akt phosphorylation	[49]
Ginsenoside Rg3	Rat TAB/Ang II induced NRVM hypertrophy	reduced oxidative stress	[50]
	Mouse TAB	improved ${\mathrm{Ca}}^{2+}$ homeostasis	[51]
Ginsenoside Rd	Mouse TAB/PE induced NRVM hypertrophy	decreased oxidative stress/MAPK signaling/inflammatory response	[52]

Table represents main findings of the specific study. See text for details. Definitions of abbreviated terms as follows: AGE, advanced glycation end products; Akt, protein kinase B; Ang II, angiotensin II; CAL, coronary artery ligation; CREB, cyclic AMP response element-binding protein; DCM, dilated cardiomyopathy; ET-1, endothelin 1; HIF-1, hypoxia-inducible factor-1; FA, fatty acid; MAPK, mitogen activated protein kinase; NE, norepinephrine; NFAT, nuclear factor of activated T cells; NF-κB, nuclear factor kappa-light-chain-enhancer of activated B cells; NHE1, sodium hydrogen exchange isoform 1; NO, nitric oxide; NRVM, neonatal rat ventricular myocytes; PE, phenylephrine; PGF2α, prostaglandin F2 alpha; PKA, protein kinase A; PPARα, proliferator-activated receptor alpha; RhoA/ROCK, Ras homolog gene family, member A/Rho-associated, coiled-coil containing protein kinase; RVH, right ventricular hypertrophy; STAT3, signal transducer and activator of transcription 3; STZ, streptozotocin; TAB, thoracic aorta banding; TGF-β/Smad, transforming growth factor beta/Smad protein; VEGF, vascular endothelial growth factor; ISO, isoproterenol; GATA-4, GATA-binding protein 4.

## 3. Ginkgo as an Antihypertrophic Agent and as a Potential Treatment for Heart Failure

In addition to ginseng as a potential ancient therapeutic tool, ginkgo (*ginkgo biloba*) has received substantial attention, particularly recently, as a potential medicinal plant therapeutic for numerous medical disorders including heart disease. Ginkgo is an ancient tree originally grown and cultivated in China whose leaf extracts have been shown to exert numerous salutary effects owing primarily to their antioxidant properties due to the presence of high amounts of flavonoids and terpenoids. However, the chemistry of ginkgo leaves is extremely complex as these contain a plethora of bioactive compounds (as was also seen for ginseng) potentially contributing to ginkgo’s therapeutic benefit. Indeed, with respect to flavonoids alone more than a hundred different flavonoid structures have been identified existing as either aglycones, glycosides or dimeric forms referred to as bioflavonoids [53, 54, 55]. As reviewed recently [55], there is substantial variability in the identification of different bioflavonoids which reflects extraction procedure as well as the part of the tree from which the extraction was made.

### Ginkgo Extract Constituents as Antihypertrophic Factors

As was seen for ginseng, ginkgo has been used as a Chinese medicine likely for thousands of years. Recently, substantial attention has been paid to the use of ginkgo extract for the treatment of dementia and cognitive impairment although the cardiovascular benefit of ginkgo has been known for some time, at least based on animal experimental studies. Thus, the ginkgo leaf extract commonly referred to as extract of ginkgo biloba (EGB) 761 has been shown to exert cardio-protection as evidenced by reduced reperfusion-induced arrhythmias [56] as well as reducing postischemic reperfusion injury and enhancing functional recovery, possibly via its antioxidant properties [57, 58, 59, 60]. However in one study no benefit of EGB 761 was evident when administered to rats followed by *ex vivo* ischemia and reperfusion unless combined with a platelet-activating factor antagonist at which time a synergistic protection was observed in terms of improved recovery of cardiac function and a reduced incidence in arrythmias [61]. The beneficial effects of ginkgo against myocardial ischemic and reperfusion injury have been demonstrated in the clinical setting in patients undergoing coronary artery bypass grafting which was proposed to reflect an inhibition of free radical production rather than free radical scavenging [62] although scavenging of nitric oxide and a reduction in pro-apoptotic signaling have also been proposed as potential mechanisms underlying cardio-protection of the ischemic and reperfused heart by ginkgo [63, 64].

While evidence for cardioprotective effects of ginkgo extract is extensive and has been known for decades, much less is known concerning any potential direct anti-remodeling effects of ginkgo unrelated to a protective influence of the ischemic and reperfused myocardium. One of the first pieces of evidence suggesting other beneficial effects of ginkgo, in addition to cardio-protection *per se* was initially presented by Timioğlu* et al*. [65] who showed that EGB 761 reduced the severity of the cardiomyopathy associated with doxorubicin (also known as adriamycin) administration to rats, an effect similarly demonstrated in mice through a mechanism involving diminished oxidative stress and lipid peroxidation [66]. More recent data suggest that the protective effect of ginkgo extract against doxorubicin-induced cardiotoxicity may involve an inhibition of mitochondrial-dependent pro-apoptotic signaling as well as reducing pro-inflammatory factors [67, 68].

In terms of antioxidant-dependent protection against doxorubicin-induced cardiotoxicity, similar antioxidant-dependent protective effects were also seen in rats treated with isoproterenol [69]. Indeed, isoproterenol-induced cardiac hypertrophy was one of the first models used to test the potential antihypertrophic effect of ginkgo. Thus, ginkgo extract normalized cholinergic and adrenergic receptor expression in rats treated for 8 days with isoproterenol and inhibited pathological myocardial remodeling and improved cardiac function in these animals [70]. While the precise mechanisms underlying the salutary effects of the ginkgo extract were not firmly established, the authors showed that the benefit was associated with normalization of cardiac muscarinic receptors and the nitric oxide synthase pathway [70].

One of the first studies to demonstrate a myocardial anti-remodeling effect of ginkgo leaf extract was one in which streptozotocin-induced diabetes in *${\mathrm{ApoE}}^{-/-}$* atherosclerosis-prone mice was associated with increased myocardial ER stress-associated apoptosis, fibrosis and upregulation of pro-inflammatory factors [71]. In this study, all manifestations of cardiac pathology and remodeling as well as the upregulation of pro-inflammatory and pro-apoptosis factors were attenuated by a ginkgo leaf extract [71]. Based on its anti-inflammatory properties, ginkgo was also studied to assess any potential benefit on cardiac pathology secondary to viral myocarditis, a serious cardiac inflammatory condition produced by viral infection and potentially leading to heart failure [72]. Using a mouse Coxsackievirus B3 viral myocarditis model, Wang* et al*. [73] showed that a ginkgo extract treatment for 30 days improved survival in these animals, an effect associated with reduced myocardial fibrosis and cell necrosis. The authors attributed the protective effect of ginkgo primarily on decreased expression of S100 calcium-binding protein A4 (S1004A), a profibrotic actor as well as matrix metalloproteinases (MMPs), particularly MMP-3 [73].

#### Possible Beneficial Role of Ginkgolides

Among the most studied of the ginkgo constituents particularly as related to potential therapeutic benefit, including cardiac anti-remodeling properties, are the terpene tri-lactones named ginkgolides including ginkgolide A, ginkgolide B, ginkgolide C, ginkgolide J, ginkgolide K, ginkgolide L and ginkgolide M. Among these, ginkgolide A has been proposed as a potential therapeutic for a number of pathological conditions [74]. While all ginkgolides have a cage-like molecular structure with six five-membered rings they differ in the position and the number of substituted hydroxyl groups which give these their specific letter designation and alters their biological profiles [53].

A potential mechanism for the beneficial effect of ginkgolides may reflect an anti-apoptotic effect of these constituents. Thus, although the precise mechanisms underlying the anti-remodeling effects of ginkgo are not known, part of this benefit may be due to improved mitochondrial function and reduced mitochondrial-dependent oxidative stress based on studies in which the leaf extract was added to cardiac mitochondrial preparations [75, 76] or after treatment of rats with a ginkgo leaf extract [77]. In this regard, a potentially important participant in the heart failure process is apoptosis or programmed cell death which is distinct from necrosis and does not involve a significant inflammatory response [78]. While there is evidence that apoptosis contributes to the heart failure process there still exists substantial uncertainty as to the degree of participation in the development of heart failure although apoptosis has been identified in various forms of heart failure [78], however this does not necessarily implicate a cause-and-effect relationship. Compared to necrosis, apoptosis is initiated by a well-ordered signaling cascade involving the release of cytochrome c from mitochondria into the cytoplasmic milieu resulting in the activation of proteolytic caspases. Extensive research on ginkgo-mediated inhibition of apoptosis is lacking although ginkgolide B was recently shown to suppress hydrogen peroxide-induced apoptosis in cultured H9c2 myoblasts, a finding which may or may not be relevant to the chronic myocardial remodeling process but likely of more significance to acute ischemic and reperfusion injury [79]. In that study the authors attributed the beneficial effect of ginkgolide B to activation of the PI3K/Akt/mTOR signaling pathway which resulted in increased phosphorylation of Akt and mTOR in hydrogen peroxide‑treated myoblasts [79]. A similar antiapoptotic effect was also seen with ginkgetin, which is a ginkgo leaf-derived biflavone that exerts a plethora of beneficial pharmacological properties although its potential cardiovascular benefit in terms of mitigating heart failure has not been studied [80]. However, ginkgetin has recently been shown to exert antiapoptotic effects in H9c2 myoblasts subjected to hypoxia and reoxygenation by inhibiting the caspase-3-dependent pathway resulting in reduced indices of oxidative stress and diminished upregulation of pro-inflammatory factors [81]. Similar beneficial effects of ginkgetin were found in terms of its ability to reduce toxicity in H9c2 cells treated with either hydrogen peroxide or phosgene [82].

Two recent studies examined specific ginkgolides to demonstrate potential therapeutic properties related to myocardial remodeling. In the first of these studies, ginkgolide B was shown to inhibit angiotensin II-induced hypertrophy and oxidative stress in H9c2myoblasts which was associated with increased markers of autophagy, an intracellular degradation process which improves cell survival and function by removing intracellular “debris” including misfolded proteins, damaged intracellular organelles, among many other components [83]. This stimulation of autophagy by ginkgolide B was proposed to occur via stimulation of the Sirtuin 1-FoxO1 (Forkhead box protein O1) pathway which plays an important role in the stimulation of autophagic activity [83]. Inhibition of myocardial remodeling in a mouse myocardial infarction model was recently demonstrated in animals treated with ginkgolide A. In this study mice were subjected to four weeks of left anterior ascending coronary artery ligation with or without daily ginkgolide A injection [84]. Animals treated with ginkgolide A demonstrated improved left ventricular function as assessed by echocardiography, reduced fibrosis as well as reduced hypertrophy [84]. In this same report, similar indices of hypertrophy and fibrosis in cultured neonatal rat ventricular myocytes treated with angiotensin II for 24 hours were also attenuated by ginkgolide A as was seen in the *in vivo* coronary artery ligation model [84]. The authors attributed an anti-inflammatory effect of ginkgolide A possibly via its binding to matrix metallopeptidase 9 [84]. Ginkgolide A has also been recently shown to attenuate cardiomyopathy in a mouse model of sepsis produced by lipopolysaccharide (LPS) administration as evidenced by a reduced inflammatory response, oxidative stress and apoptosis [85]. In this report the beneficial effects of ginkgolide A were attributed to its ability to prevent LPS-induced downregulation of nuclear FoxO1, a transcriptional factor responsible for increased expression of cardioprotective genes [85]. Thus, while this study as well as the study referred to above concerning ginkgolide B [83] suggest that FoxO1 upregulation is important for the beneficial effects of ginkgolides, it should be added that the role of this transcriptional factor particularly with respect to cardiac hypertrophy and related pathologies is far from completely understood and requires substantial research to delineate [86].

A summary of the effects of ginkgo and ginkgolides and their proposed underlying mechanisms is presented in Table 2 (Ref. [65, 66, 67, 68, 69, 70, 71, 73, 83, 84, 85]).

**Table 2.** **Studies demonstrating antiremodeling effects and reduction in heart failure by total ginkgo extract and individual ginkgolides in various experimental models and their proposed mechanisms of action**.

Ginkgo or ginkgolide	Experimental model	Proposed mechanism(s)	Ref
Ginkgo extract	Adriamycin treated rats	antioxidant	[65]
	Adriamycin treated mice	antioxidant	[66]
	Adriamycin treated rats	reduced apoptosis	[67]
	Adriamycin treated rats	increased NO generation antioxidant	[68]
	Isoproterenol treated rats	antioxidant	[69]
	Isoproterenol treated rats	increased NO generation	[70]
	Isoproterenol treated NRVM	improved NO generation	[68]
	STZ treated AP mice	reduced apoptosis	[71]
		reduced inflammatory response	
	Mice viral myocarditis	decreased S100A4 and MMP-3 expression	[73]
Ginkgolide B	Ang II induced H9c2	increased autophagy	[83]
Ginkgolide A	Mouse CAL	reduced inflammatory response	[84]
	Ang II induced NRVM hypertrophy	reduced inflammatory response	[84]
	LPS-induced sepsis in mice	decreased nuclear FoxO1	[85]

Table represents main findings of the specific study. See text for details. Definitions of abbreviated terms as follows: Ang II, angiotensin II; CAL, coronary artery ligation; FoxO1, Forkhead box protein O1; MMP-3, matrix metalloproteinase-3; NO, nitric oxide; NRVM, neonatal rat ventricular myocytes; S100A4, S100 calcium-binding protein A4; STZ, streptozotocin; LPS, lipopolysaccharide.

## 4. Perspectives and Future Directions

**Fig. 2.**
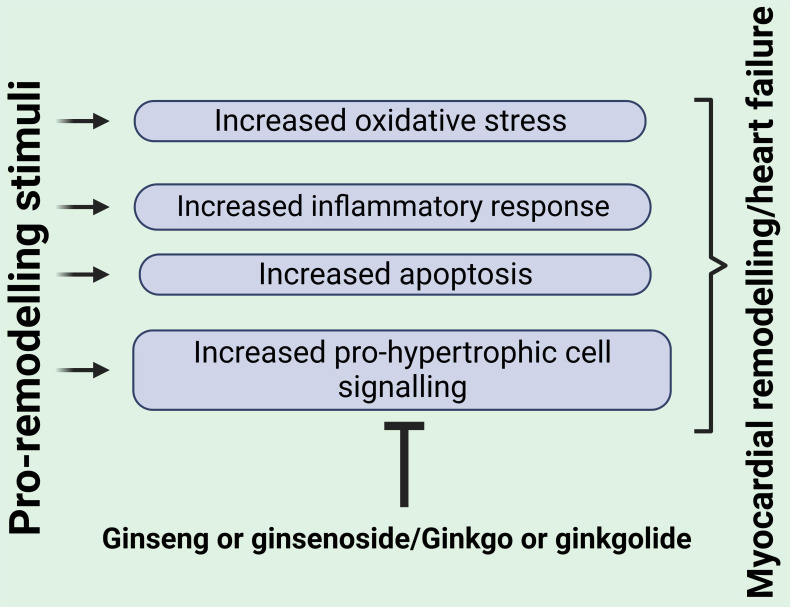
**Key mediators of myocardial remodeling and heart failure commonly targeted by both ginseng and ginkgo or their bioactive components**. See text for details. Created with BioRender.com.

In this review we present evidence for the beneficial effects of ginseng and *ginkgo biloba* as well as their active components in mitigating the myocardial remodeling process and the resultant heart failure. Indeed, traditional Chinese medicines have been used for thousands of years targeting a plethora of maladies including heart disease and as such their ability to attenuate myocardial remodeling may therefore not be surprising. One major challenge in this field is to delineate the underlying mechanism for their beneficial effects as the evidence suggests a multiplicity of cellular effects which could account for their therapeutic actions. There are several reasons for such diverse complex effects of these agents. Firstly, as is evident from this review, numerous experimental models including both *in vivo* and *in vitro* approaches have been used to study the potential beneficial effects of these agents with each model representing distinct cellular mechanisms. Moreover, even within these approaches different and specific pathological insults have been employed. As one example pertaining to *in vivo* studies, molecular and cellular mechanisms contributing to myocardial remodeling and heart failure following myocardial infarction are quite distinct from a pressure overload response produced thoracic aorta coarctation. The same argument can be applied to studies using isolated or cultured myocytes where the underlying mechanism involved in the hypertrophic response would be dictated to a large degree by the specific stimulus used. Adding to the complexity underlying our understanding of specific mechanism is the fact that the net effect of ginseng and ginkgo represents the nature of their individual constituents, particularly ginsenosides and ginkgolides, respectively. The nature of these constituents varies substantially, particularly with regards to ginseng where the types of the ginsenosides varies by ginseng species or indeed by ginseng manipulation such as that produced by heating as noted in Section 2. In addition, different ginkgolides exert different biological responses. As noted above, it is well-established that the cellular mechanisms which account for the myocardial remodeling process are multifaceted and each of these pathways represent a potential target for anti-remodeling effects of therapeutic agents including ginseng and ginkgo and their constituents (Fig. 1). However, as illustrated in Fig. 2 a number of common targets can be identified whose inhibition contribute to the beneficial effects of both ginseng- and ginkgo-related compounds, including inhibition of the inflammatory response, oxidative stress, pro-hypertrophic cell signaling as well as apoptosis. These effects would all contribute to a reduction in the myocardial remodeling process. Taken together, it can be reasonably assumed that ginseng and ginkgo as well as their respective bioactive constituents inhibit myocardial remodeling and heart failure through multiple mechanisms dictated by the nature of the protective factor (e.g., ginseng species or specific ginsenoside or ginkgolides), the experimental model to induce remodeling as well as the primary underlying mechanism underlying the remodeling process. The relative contribution of each of these mechanisms to the net beneficial effects of these agents requires further studies to delineate.

A major challenge for the future is to reinforce the concept of beneficial effects of natural products such as ginseng or ginkgo in the clinical setting based on well-designed clinical trials. At present such evidence is lacking as the use of these products for the treatment of heart failure is generally based on rather small clinical trials. As such, well-designed, randomized and placebo-controlled Phase 3 clinical trials are needed although it should be appreciated that establishing phase 3 clinical trials is challenging on many fronts particularly in view of the very large financial costs involved.

Another important issue to consider when administering natural products for the treatment of heart failure is the potential for drug interactions particularly as many patients with heart failure are already treated with a rather large number of medications. Although this concern has been previously recognized [87, 88], nonetheless, interaction of herbal medications with standard pharmacological agents remains an underexplored area of clinical research with a paucity of information related to this very important issue. Specifically with regards to ginkgo, it has been suggested that the leaf extract EGB 761 is generally devoid of potential drug interactions when consumed at a dose of less than 240 mg/day [89]. It should be noted that interaction between herbal medications may not necessarily reflect untoward consequences as there is some evidence of synergistic interaction to improve symptoms of heart failure as has been shown in a small group of patients administered both digoxin and red ginseng who demonstrated significantly better hemodynamic function than that seen in patients on either agent alone [90]. The concept of ginseng as an effective adjuvant to standard heart failure treatment has been reinforced in a recent report analyzing 28 publications where ginseng-containing compound were co-administered with standard therapies and which showed that addition of these compounds enhanced benefit to that seen with standard therapy alone [91]. However, as noted by the authors, analyses of these studied should be done cautiously particularly in view of the small subject group recruited to each study [91]. Clearly, substantial research is required in this area in order to fulfil the potential of ginseng, ginkgo and indeed other herbal medications as effective adjunctive therapies for the treatment of heart failure.

## 5. Conclusions

Traditional Chinese medicines such as ginseng and ginkgo, discussed here, as well as others have a long history of use for the treatment of cardiovascular diseases and other conditions especially in Asian societies. These phytochemicals have, in general, been shown to exert a plethora of beneficial effects particularly in experimental studies. However, convincing clinical data are lacking, a situation which can be rectified by carrying out well-controlled clinical trials. In addition to the paucity of clinical data, introducing ginseng or ginkgo for widespread general use for the treatment of heart failure represents a major challenge due to insufficient data concerning many factors including possible untoward effects, interactions with other medications and a clear understanding of underlying mechanisms of action. Yet, we believe that these products hold promise and we hope that future research will address these issues and justify their addition to the armamentarium of drugs for the treatment of heart failure.
